# Factor structure of the Maslach Burnout Inventory Human Services Survey in Spanish urgency healthcare personnel: a cross-sectional study

**DOI:** 10.1186/s12909-022-03666-3

**Published:** 2022-08-12

**Authors:** Carles Forné, Oriol Yuguero

**Affiliations:** 1 Department of Health Economics and Outcomes Research, Heorfy Consulting, Lleida, Spain; 2 Department of Basic Medical Sciences, University of Lleida, Av. Alcalde Rovira Roure 80; Biomedicina building, module II, floor 4, 25198 Lleida, Spain; 3 Emergency Service, University Hospital Arnau de Vilanova, Av. Alcalde Rovira Roure 80, 25198 Lleida, Spain; 4 Urgency and Emergency Multidisciplinary Research Group, Lleida Institute for Biomedical Research Dr. Pifarré Foundation (IRBLleida), Lleida, Spain

**Keywords:** Burnout, Psychological, Factor Analysis, Statistical, Maslach Burnout Inventory – Human Services Survey, Emergencies, Psychometrics

## Abstract

**Background:**

The Maslach Burnout Inventory (MBI) is an instrument commonly used to evaluate burnout syndrome. The goal of the present study was to assess the internal reliability and the performance of the items and the subscales of the MBI-HSS (the version for professionals working in human services) by validating its factorial structure in Spanish urgency healthcare personnel.

**Methods:**

Cross-sectional study including 259 healthcare emergency professionals (physicians and nurses) in the Spanish health region of Lleida and the Pyrenees. Burnout was measured using the Spanish validated version of the MBI-HSS. Internal reliability was estimated using Cronbach’s alpha coefficient. The sampling adequacy was assessed using the Kaiser-Meyer-Olkin measure along with the Bartlett’s test of sphericity. A principal axis exploratory factor analysis with an oblique transformation of the solution and a confirmatory factor analysis with maximum likelihood estimation were performed. Goodness-of-fit was assessed by means of the chi-square ratio by the degrees of freedom, the standardized root mean square residual (SRMR), the root mean square error of approximation (RMSEA), the Tucker-Lewis Index (TLI) and the comparative fit index (CFI).

**Results:**

The three subscales showed good internal reliability with Cronbach’s alpha coefficients exceeding the critical value of 0.7. Exploratory factor analysis revealed five factors with eigenvalues greater than 1. Nevertheless, confirmatory factor analysis showed a relatively satisfactory fit of the three-factor structure (χ^2^/df = 2.6, SRMR = 0.07, RMSEA = 0.08, TLI = 0.87, CFI = 0.89), which was improved when several items were removed (χ^2^/df = 1.7, SRMR = 0.04, RMSEA = 0.05, TLI = 0.97, CFI = 0.98).

**Conclusions:**

Although it is necessary exploring new samples to get to more consistent conclusions, the MBI-HSS is a reliable and factorially valid instrument to evaluate burnout syndrome in health professionals from the Spanish emergency services.

## Background

In recent decades, professional burnout among health professionals has become a topic of great interest for researchers around the world; partly due to the great burden on the national health systems and on society in general. The effects resulting from burnout have proven to cause issues in workers’ physiological (e.g., cardiovascular diseases) and psychological (e.g., mental disorders) conditions, and affecting their performance at work (e.g., increased dissatisfaction, absenteeism and presenteeism) [1].

Introduced by Freudenberger, staff burnout is a state of mental, emotional, and physical exhaustion that occurs as a result of overwhelming demands, chronic stress, or job dissatisfaction [2]. Maslach later provided a comprehensive definition of the term involving emotional exhaustion (EE), depersonalization (DP), and a reduced sense of personal accomplishment (PA) that can occur among individuals who work with people in some way [3]. Thus, EE assesses feelings of being emotionally overwhelmed and exhausted by one’s work; DP measures a callous and impersonal response toward recipients of one’s service, care or treatment; and PA assesses feelings of competence and successful achievement in one’s work. The definition provided by Maslach led to the subsequent identification of these three main dimensions of burnout evaluated through the Maslach Burnout Inventory (MBI) [4, 5]. Other several instruments exist to measure job-related burnout in human service professionals. Among these are the Staff Burnout Scale [6, 7] and the Burnout Measure [8]. By far, the MBI is the worldwide leading instrument for assessing burnout. As Schaufeli et al. [9] pointed out, the success of the MBI may lie in the work of Perlman and Hartman [10], who after a review of more than 48 definitions of the burnout syndrome concluded that burnout should be defined as “a response to chronic emotional stress with three components: (a) emotional and/or physical exhaustion, (b) lowered job productivity, and (c) overdepersonalization.” This definition was very similar to the one proposed by Maslach and Jackson [4, 5] as a result of factoring the MBI, and probably generalized its use and acceptance.

Currently, there are three versions of the MBI: the General Survey (MBI-GS), used for workers in general; the Educators Survey (MBI-ES), used in the educational area; and the Human Services Survey (MBI-HSS), used for the health services [11]. Several studies have been published carrying out exploratory and/or confirmatory analyses of the factorial structure of the MBI in different professional groups [12]. Although the studies carried out with health professionals have focused mainly on nursing professionals, both emergency and non-emergency nursing care [13, 14], even exploring differences between countries [15], there are also some studies including physicians [16], and including all emergency professional profiles [17]. But we are not aware of any study exploring the factorial validity of the MBI-HSS in a cohort of urgency healthcare professionals in Spain.

In this study, we aimed to assess the internal reliability and the performance of the items and the subscales of the MBI-HSS by validating its factorial structure in Spanish urgency healthcare personnel.

## Methods

This is a cross-sectional observational study conducted in the health region of Lleida and the Pyrenees. In this health region, there are 5 public hospitals and 3 private hospitals. There are also 12 continuous care centers in primary care and 6 mobile units of outpatient emergencies.

All medical and nursing professionals of the health region who work in public emergency care centers were contacted by emails in two different calendar periods more than 4 years apart. Participants who voluntarily agreed to participate completed an anonymous survey on burnout between May and September 2016 (first wave) and/or between November 2020 and January 2021 (second wave). At the time of the survey there were 245 (first wave) and 267 (second wave) professionals working in the centers described above, and a response rate of 40.8 and 59.6% was reached, respectively. Data were anonymized to ensure confidentiality. The study design and methods were previously described [18].

### Burnout measure

Burnout was measured using the MBI-HSS [4] in the version validated in Spanish [19] and previously used in other studies [18, 20, 21]. The MBI-HSS is an instrument of 22 Likert items of 7 points on feelings related to work. Respondents rate how often they experience these feelings on a 7-point Likert scale from 0 (never) to 6 (every day). The MBI includes 3 subscales or dimensions: emotional exhaustion (EE), depersonalization (DP), and personal accomplishment (PA). Higher item scores in EE and DP and low in PA correspond to high levels of burnout. The scale used in this study was described in previous research from our group [18].

### Statistical analysis

Characteristics of the study sample were described by means of frequencies and percentages. Responses of the MBI-HSS were summarized obtaining the median and the percentiles of the 25% and the 75%. Subscales scores were described calculating means and standard deviations and pairwise correlations. The reliability was assessed using the Cronbach alpha.

In order to examine the interrelationships between the items and to confirm the latent structure of the MBI-HSS, an exploratory factor analysis (EFA) was performed. The sampling adequacy for use in the EFA was assessed by means of the Kaiser-Meyer-Olkin (KMO) measure along with the Bartlett’s test of sphericity (KMO ≥ 0.5 and *p* < 0.001 show sampling adequacy). We carried out a principal axis factor analysis with an oblique transformation of the solution. Initially, factors were extracted based on eigenvalues greater than 1, next the number of factors was limited to three, as the original factor analysis of the MBI-HSS.

Next, a confirmatory factor analysis (CFA) with maximum likelihood estimation was conducted to test the underlying factor structure based on the EFA findings. Four models were fitted according to the criteria for the inclusion of the items in the analysis: (M1) those with standardized factor loadings greater than 0.4 (as the original factor analysis of the MBI-HSS); (M2) greater than 0.3; (M3) greater than 0.5; and (M4) greater than 0.6. Several goodness-of-fit indicators were reported as the use of multiple fit indices provides a more holistic view: the relative chi-square (χ^2^/df), the standardized root mean square residual (SRMR), the root mean square error of approximation (RMSEA), the Tucker-Lewis Index (TLI), and the comparative fit index (CFI). The χ^2^/df depends both sample size and the chi-square statistic itself, and as a consequence, different researchers have recommended using ratios as low as 2 or as high as 5 to indicate a reasonable fit. Recommended thresholds to conclude that there is a relatively good fit between the hypothesized model and the observed data are 0.95 for TLI and CFI; 0.08 for SRMR; and 0.06 for RMSEA [22].

The R programming language [23] and the RStudio environment [24] were used for the data analysis. The cronbach function of the multilevel package [25] was used to obtain the Cronbach alpha; the bart_spher and the KMOS functions of the REdaS package [26, 27] were used to assess the sampling adequacy for factor analysis; the fa function of the psych package [28] was used to perform the EFA; and the cfa and the fitMeasures functions of the lavaan package [29, 30] were used to perform the CFA and to calculate the goodness-of-fit measures, respectively.

## Results

### Sample characteristics

Characteristics of the study sample are shown in Table 1. Most of the participants were women (66.8%) aged between 30 and 49 (58.7%). Slightly more than half of the participants were physicians (51.7%), had work practice experience of 10 or more years (55.2%) and were working in a hospital of second level (51.0%). Almost half of the participating urgency healthcare personnel had other occupation (45.1%).

**Table 1:** Characteristics of the study sample (*N* = 259)

Characteristic	n (%)
Sex	
*Men*	86 (33.2%)
*Women*	173 (66.8%)
Age group	
*< 30*	42 (16.2%)
*30–39*	84 (32.4%)
*40–49*	68 (26.3%)
*50–59*	56 (21.6%)
*60+*	9 (3.47%)
Profession	
*Nurse*	125 (48.3%)
*Physician*	134 (51.7%)
Resident	
*No*	245 (94.6%)
*Yes*	14 (5.41%)
Level of care	
*Hospital of 2nd level*	132 (51.0%)
*Outpatient emergency care*	35 (13.5%)
*Primary emergency care*	28 (10.8%)
*Regional hospital*	64 (24.7%)
Type of center	
*Private*	13 (5.02%)
*Public*	246 (95.0%)
Worked years	
*< 5*	65 (25.1%)
*5–10*	51 (19.7%)
*10–15*	58 (22.4%)
*16–20*	34 (13.1%)
*21+*	51 (19.7%)
Other occupation	
*No*	141 (54.9%)
*Yes*	116 (45.1%)

### Descriptive and reliability of the MBI-HSS

Participants responded all the items of the MBI-HSS. The descriptive statistics of their responses and of the three subscales are shown in Table 2. The reliability estimates calculated on this sample of urgency healthcare personnel showed adequate internal consistency for each of the three MBI-HSS scales, with all values above the generally accepted threshold of 0.7, and were very similar to those published by Maslach et al. [4].

**Table 2:** Descriptive statistics of the MBI-HSS (*N* = 259)

Item number		Median (P25, P75)			
1		3 (1, 5)			
2		5 (3, 5)			
3		2 (1, 5)			
4		5 (5, 6)			
5		1 (0, 2)			
6		4 (2, 5)			
7		5 (4, 6)			
8		3 (1, 5)			
9		5 (4, 6)			
10		1 (0, 4)			
11		2 (1, 4)			
12		5 (4, 5.5)			
13		2 (1, 4)			
14		4 (2, 5)			
15		0 (0, 1)			
16		1 (1, 3)			
17		5 (4, 6)			
18		5 (4, 6)			
19		5 (4, 6)			
20		0 (0, 2)			
21		5 (3, 5)			
22		1 (1, 3)			
			**Correlations**		
**Subscales**	**Mean (SD)**	**Cronbach’s α**	**EE**	**DP**	**PA**
Emotional exhaustion (EE)	25.8 (12.6)	0.908	1	0.602	−0.357
Depersonalization (DP)	8.62 (6.17)	0.730	–	1	−0.317
Personal accomplishment (PA)	36.7 (6.96)	0.807	–	–	1

*P25* percentile of the 25%, *P75* percentile of the 75%, *SD* standard deviation*, EE* emotional exhaustion, *DP* depersonalization, *PA* personal accomplishment

### Exploratory factor analysis

The KMO measure and the Bartlett’s test of sphericity indicated that the data were suitable for factor analysis (KMO = 0.898, *p* < 0.001). Analysis of the eigenvalues suggested that five factors should be extracted with values above 1 (8.335, 2.992, 1.499, 1.152, 1.014), that explained 51.4% of the total variance. Table 3 shows the factor loadings for this solution. According to the criterion of assigning an item to the factor in which it presented a factor loading greater than 0.4: the first principal axis factor (F1) accounted for 20.6% of the variance and combined seven of the nine items of the EE subscale (items 1, 2, 3, 6, 8, 13 and 14); the second principal axis factor (F2) accounted for 7.9% of the variance and combined four of the eight items of the PA subscale (items 4, 7, 17 and 21); the third principal axis factor (F3) accounted for 9.6% of the variance and combined three of the five items of the DP subscale (items 5, 10 and 15); the fourth principal axis factor (F4), accounted for 8.4% of the variance, and combined three items of the PA subscale (items 9, 12 and 19); and the fifth principal axis factor (F5) accounted for 4.9% of the variance and combined only two items, the items 16 and 22, included in the EE and DP subscales, respectively.

**Table 3:** Exploratory Factor Analysis of the MBI-HSS (N = 259)

Item number	First Exploratory Factor Analysis					Second Exploratory Factor Analysis		
	F1	F3	F4	F2	F5	F1	F2	F3
1	**0.90**	0.02	0.00	0.05	−0.04	**0.88**	0.03	0.00
2	**0.78**	−0.10	0.00	−0.08	0.04	**0.79**	−0.04	−0.11
3	**0.80**	0.04	−0.08	0.05	0.03	**0.85**	0.00	0.02
4	0.04	−0.29	−0.01	**0.45**	0.30	0.28	**0.48**	−0.23
5	0.09	**0.70**	0.01	−0.06	0.00	0.08	−0.08	**0.68**
6	**0.40**	0.09	−0.02	−0.10	0.24	**0.54**	−0.03	0.10
7	0.11	0.04	0.17	**0.56**	−0.14	0.07	**0.56**	0.04
8	**0.85**	0.07	−0.04	0.00	−0.02	**0.85**	−0.04	0.04
9	0.17	−0.20	**0.50**	0.28	0.06	0.11	**0.66**	−0.13
10	0.01	**0.70**	−0.08	0.08	0.01	0.06	−0.03	**0.66**
11	0.18	0.36	−0.07	0.08	0.20	0.33	0.06	0.37
12	−0.21	0.01	**0.55**	0.07	−0.01	−0.35	**0.46**	0.06
13	**0.66**	0.16	−0.14	0.09	0.03	**0.72**	−0.03	0.13
14	**0.65**	−0.03	0.22	−0.22	0.13	**0.62**	0.02	0.01
15	0.04	**0.56**	0.15	−0.14	0.10	0.05	−0.01	**0.56**
16	0.10	0.07	−0.05	−0.11	**0.54**	**0.42**	0.02	0.12
17	−0.08	−0.01	0.14	**0.60**	−0.14	−0.11	**0.58**	−0.01
18	−0.16	−0.16	0.36	0.30	0.12	−0.13	**0.59**	−0.09
19	−0.07	0.06	**0.72**	0.10	−0.02	−0.24	**0.59**	0.11
20	0.26	0.23	−0.39	0.19	0.26	**0.54**	−0.07	0.20
21	−0.11	−0.03	0.11	**0.55**	0.16	0.05	**0.62**	0.01
22	0.01	0.29	0.10	0.02	**0.43**	0.26	0.19	0.32
SS loadings	4.538	2.109	1.858	1.734	1.069	5.306	2.771	1.933
Variance explained (%)	20.6	9.6	8.4	7.9	4.9	24.1	12.6	8.8

Factor loadings greater than 0.4 in bold

*SS* sum of squared

The interpretability of the five-factor model seemed to be complicated, since it split the PA into two different factors, and part of the EE and the DP were in a fifth factor. So, the three-factor model was explored in order to check the degree of adjustment of our data to the subscale composition provided by the MBI-HSS authors [4]. This model explained 45.5% of the total variance. Table 3 also shows the factor loadings for this solution. When compared with the five-factor model, it can be seen that items 9, 12 and 19 of the previous F4 factor were included into F2; and the item 16 of the previous F5 factor were included into F1; the item 22 did not show loadings higher than 0.4 in any factor of the three-factor solution, although it had the highest loading in F3. Both five-factor and three-factor models have almost equal loadings for their first two principal axis factors. Since the structure of the three-factor model was practically the same as the original subscale composition, the extracted factors could be interpreted as F1 = EE, F2 = PA and F3 = DP.

### Confirmatory factor analysis

**Fig. 1:**
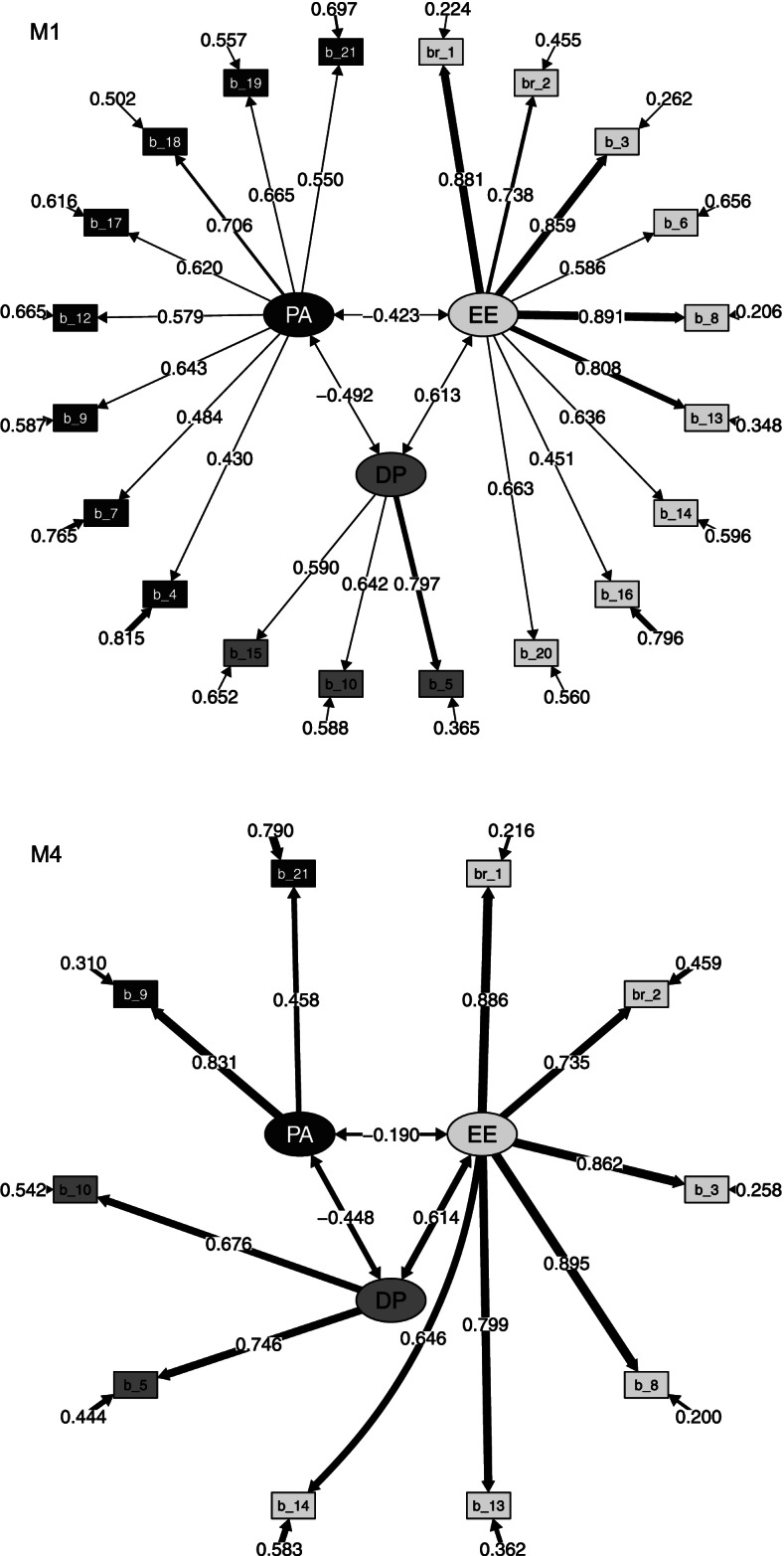
Standardized parameter estimates for the factor structure of the MBI-HSS according to models M1 and M4. Squares indicate the items on the MBI-HSS, circles represent the 3 latent factors associated with the subscales. The grayscale fill identifies the factor loadings proposed by Maslach et al. [4] Parameter estimates (factor loadings, correlations among factors, and residual variances) are based on a sample of 259 healthcare emergency professionals (physicians and nurses) in the Spanish health region of Lleida and the Pyrenees

The factorial structure of the MBI-HSS was tested using CFA (Table 4). The first analysis (M1) examined the three-factor model found in EFA (Table 2). This model replicated the structure proposed by the original Maslach’s model, except that items 11 and 22 were not included in any subscale. Figure 1 shows the path diagram with standardized estimates of the model M1. Since M1 showed a relatively but not completely satisfactory fit, the model was revised to achieve a better goodness of fit by varying the number of items in each factor.

**Table 4:** Confirmatory Factor Analysis of the MBI-HSS (N = 259)

Standardized regression weights (factor loadings)	M1		M2		M3		M4	
	Estimate	***p***-value	Estimate	***p***-value	Estimate	***p***-value	Estimate	***p***-value
EE → B1	0.881	< 0.001	0.880	< 0.001	0.883	< 0.001	0.886	< 0.001
EE → B2	0.738	< 0.001	0.737	< 0.001	0.738	< 0.001	0.735	< 0.001
EE → B3	0.859	< 0.001	0.862	< 0.001	0.861	< 0.001	0.862	< 0.001
EE → B6	0.586	< 0.001	0.585	< 0.001	0.579	< 0.001		
EE → B8	0.891	< 0.001	0.889	< 0.001	0.892	< 0.001	0.895	< 0.001
EE → B13	0.808	< 0.001	0.806	< 0.001	0.807	< 0.001	0.799	< 0.001
EE → B14	0.636	< 0.001	0.634	< 0.001	0.635	< 0.001	0.646	< 0.001
EE → B16	0.451	< 0.001	0.453	< 0.001				
EE → B20	0.663	< 0.001	0.667	< 0.001	0.658	< 0.001		
DP → B5	0.797	< 0.001	0.751	< 0.001	0.800	< 0.001	0.746	< 0.001
DP → B10	0.642	< 0.001	0.686	< 0.001	0.640	< 0.001	0.676	< 0.001
DP → B11			0.401	< 0.001				
DP → B15	0.590	< 0.001	0.573	< 0.001	0.588	< 0.001		
DP → B22			0.412	< 0.001				
PA → B4	0.430	< 0.001	0.444	< 0.001				
PA → B7	0.484	< 0.001	0.500	< 0.001	0.474	< 0.001		
PA → B9	0.643	< 0.001	0.653	< 0.001	0.635	< 0.001	0.831	< 0.001
PA → B12	0.579	< 0.001	0.448	< 0.001				
PA → B17	0.620	< 0.001	0.627	< 0.001	0.644	< 0.001		
PA → B18	0.706	< 0.001	0.697	< 0.001	0.711	< 0.001		
PA → B19	0.665	< 0.001	0.656	< 0.001	0.649	< 0.001		
PA → B21	0.550	< 0.001	0.562	< 0.001	0.556	< 0.001	0.458	< 0.001
EE → B11			0.243	0.002				
EE → B12			−0.280	< 0.001				
**Goodness-of-fit measures**								
χ^2^/df	2.551	< 0.001	2.399	< 0.001	2.421	< 0.001	1.724	0.007
SRMR	0.074		0.069		0.062		0.038	
RMSEA (95% CI)	0.077 (0.068, 0.087)		0.073 (0.065, 0.082)		0.074 (0.063, 0.085)		0.053 (0.028, 0.076)	
TLI	0.874		0.871		0.906		0.975	
CFI	0.889		0.886		0.920		0.982	
**Correlations**								
EE – DP	0.613	< 0.001	0.640	< 0.001	0.610	< 0.001	0.614	< 0.001
EE – PA	−0.423	< 0.001	−0.372	< 0.001	− 0.400	< 0.001	− 0.190	0.015
DP – PA	−0.492	< 0.001	−0.436	< 0.001	− 0.475	< 0.001	−0.448	< 0.001

The four models fitted are: (M1) including the items with standardized factor loadings of the EFA greater than 0.4; (M2) greater than 0.3; (M3) greater than 0.5; and (M4) greater than 0.6. B1, …, B22 are the MBI-HSS items

*EE* emotional exhaustion, *DP* depersonalization, *PA* personal accomplishment, *SRMR* standardized root mean square residual, *RMSEA* root mean square error of approximation, *CI* confidence interval, *TLI* Tucker-Lewis Index, *CFI* comparative fit index

Increasing the number of items in each factor (i.e., including items showing standardized factor loadings of the EFA greater than 0.3) showed a very slight and insufficient improvement in some goodness-of-fit indices. In the model M2, there were two cross-loadings: item 11 was linked with both EE (F1) and DP (F3), and item 12 was linked with both EE (F1) and PA (F2) as corresponding loadings were greater than 0.3. However, standardized estimates showed that items 11 and 12 had lower loading on factor EE.

Decreasing the number of items in each factor (i.e., including items with standardized factor loadings of the EFA greater than 0.5 [M3] and 0.6 [M4]) showed better goodness-of-fit, especially the simplest model [M4], with all of the indices meeting the minimum commonly accepted criteria: χ^2^/df = 1.72, SRMR = 0.04, RMSEA = 0.05, TLI = 0.97 and CFI = 0.98. The model M4 included 10 items of the MBI-HSS: 6 were loaded on the EE factor, 2 items were loaded on the DP factor, and also 2 items were loaded on the PA factor. Figure 1 shows the path diagram with standardized estimates of the model M4.

## Discussion

The results obtained in the present study show an adequate internal consistency of the MBI-HSS for health professionals in emergency services for the three factors: especially in EE and PA, with values higher than the recommended cut-off of 0.8 for most empirical studies [31], and a bit lower in DP, although exceeding the traditionally recommended cut-off of 0.7 [32]. Our figures were very similar to the internal consistency coefficients reported by Maslach et al. [4] (0.90 for EE, 0.79 for DP, and 0.71 for PA). Similar results were also found in other studies [16, 33–36], even in those carried-out in other countries [14, 15, 17, 37–42], indicating that the scale maintains its validity despite cross-cultural differences [12, 15].

Although EFA showed a bit complex factorial structure splitting the MBI subscales in different factors, we think that our findings also confirm the three-dimensional factorial structure underlying the MBI-HSS as proposed by the original Maslach’s model [4], as well as in other studies in which the factorial structure found as in the original version was maintained [15–17, 33, 36, 40, 42]. Previous studies have questioned the validity of some of the items. Item 12, designed to measure PA, has been shown to be cross-loaded on the EE factor; similarly, item 16 is an EE item with significant loadings on the DP subscale [4]. Apart from items 12 and 16, similar problems have also been observed with other items, such as 6 and 22, which did not load on the expected factor or did not load on any factor [12]. Despite confirming the three-factor structure, our findings from CFA were more in line with studies that suggested that the initial three-factor structure could better fit the data if several items were excluded [14, 36, 39–41].

With the initial model (M1), a relatively good fit was obtained, measured with the SRMR, according to the “rule of thumb” of the conventional cut-off value of 0.08 [22]; but the other indices did not reach the established thresholds, although they were reasonably close. When comparing M1 with more complex or simpler specifications in terms of items considered, interesting findings were observed. Although increasing the number of items led to a better fit (M2), the improvement in the goodness-of-fit indices was marginal. In view of the results obtained with the more complex model (M2), simpler models (M3 and M4) were adjusted. Interestingly, removing a few items from the M1 model (item 16 from EE; items 4 and 12 from PA) did not improve some of the goodness-of-fit indices: M3 improved all indices without reaching the minimum usually cut-offs established as thresholds of good fit [22].

The simplest model (M4) deleting even more items compared to M3 (items 6 and 20 from EE; item 15 from DP; items 7, 17, 18 and 19 from PA) achieved satisfactory goodness-of-fit values, all of them reaching the recommended criteria. Still, these results do not justify a redefinition of valid items nor an excessive scale shortening.

Although some items are known to be ambiguous [13, 14], there is no consensus on the items that may be excluded from the scale. In this sense, and given that factorial loads of the items could be related to the sample characteristics or the cultural factors [12, 15], different studies have shown that some items end up being removed [14, 36, 39, 40].

We would like to point out that it is very important to develop and make available tools to be able to assess the degree of burnout in health professionals. At a time of change in the health system, it is important to have objective instruments to be able to evaluate the impact of the interventions that can be taken to reduce and decrease burnout.

### Limitations

This study has several limitations. It is possible that some professional answered the survey in both waves. Although we are aware that analyzing the answers as independent carries a risk of underestimating the standard errors, unfortunately, data anonymization system did not allow to identify which professionals have responded to each of the surveys. However, we think that the more than 4 years elapsed between both waves of the survey minimized the impact of the within-individual correlation on results, as total variability would be mainly due to variability between individuals rather than within-individual variability. The representativeness of the sample, belonging to a single health region, limits the results to be generalized to emergency professionals from any territory in Spain, given that the health system in our country is decentralized. This means that the management of health centers and the conditions and characteristics of emergency professionals vary depending on each Autonomous Community of Spain. Moreover, differences in population and work environment may have different requirements and pressures on emergency professionals. However, generalization could be possible as the professionals’ competency is similar throughout the country. The behavior of a scale in one sample does not ensure the same behavior in other samples. However, this study provides valuable information for understanding the syndrome in this particular region. In addition, the available sample size has not allowed exploring more complex models, incorporating correlated error variances between items.

On the other hand, given that the questionnaire is self-administered, and that the participants were interviewed online, the responses may be biased. There may be other sources of bias, such as the fact that the questionnaire was answered by volunteer participants, so it is possible that those who answered the survey were more likely to experience burnout or overestimate their symptoms; although the opposite effect could also exist, and those with higher levels of burnout were not willing to answer questions that they might consider too sensitive. In addition, the answers were collected in different calendar periods, so there could be different temporal determinants in the professional burnout of the participants, which modified their personal feelings.

Finally, the choice of the goodness-of-fit indices could have conditioned the interpretation of the results. In this sense, widely and commonly used absolute and relative indices were chosen, which have also shown to be robust to the estimation technique and the sample size [43].

## Conclusions

Although it is necessary exploring new samples to get to more consistent conclusions, the present study confirms the factorial validity of MBI-HSS instrument, and that its scales present internal consistency to evaluate burnout syndrome in health professionals from the Spanish emergency services.

## Data Availability

The datasets generated and analyzed during the current study are not publicly available due to ethical restrictions, but are available from the corresponding author on reasonable request.
